# Genetic Mosaicism in a Group of Patients With Cornelia de Lange Syndrome

**DOI:** 10.3389/fped.2019.00203

**Published:** 2019-05-15

**Authors:** Natalia Krawczynska, Jolanta Wierzba, Bartosz Wasag

**Affiliations:** ^1^Department of Biology and Medical Genetics, Medical University of Gdańsk, Gdańsk, Poland; ^2^Laboratory of Clinical Genetics, University Clinical Centre, Gdańsk, Poland; ^3^Department of General Nursery, Medical University of Gdańsk, Gdańsk, Poland

**Keywords:** Cornelia de Lange Syndrome, CdLS, genetic mosaicism, deep sequencing, RNA analysis

## Abstract

**Background:** Cornelia de Lange Syndrome (CdLS) is a heterogeneous disorder. Diverse expression of clinical symptoms can be caused by a variety of pathogenic variants located within the sequence of different genes correlated with the cohesin complex.

**Methods:** Sixty-nine patients with confirmed clinical diagnosis of CdLS were enrolled in the study. Blood and buccal swab samples were collected for molecular studies. Mutational analysis was performed using the Next Generation (deep) Sequencing (NGS) covering 24 genes. In addition, the MLPA technique was applied to detect large rearrangements of *NIPBL*.

**Results:** MLPA and NGS analysis were performed in 66 (95,7%) and 67 (97,1%) patients, respectively. Large rearrangements of *NIPBL* were not identified in the studied group. Germline pathogenic variants were detected in 18 (26,1%) patients. Fourteen variants (20,3%) were identified in *NIPBL*, two (2,9%) in *SMC1A*, and two (2,9%) in *HDAC8*. In total, 13 (18,8%) buccal swabs were suitable for deep sequencing. Mosaic variants were found in four (30,8%; 4/13) patients negative for germline alterations. Three mosaic substitutions were detected in *NIPBL* while one in *KMT2A* gene.

**Conclusions:** Comprehensive and sensitive molecular techniques allow detecting novel pathogenic variants responsible for the molecular basis of CdLS. In addition, molecular testing of different tissues should be applied since such an approach allows detect mosaic variants specific for a subgroup of CdLS patients. Finally, to test possible pathogenicity of intronic variants, RNA analysis should be conducted.

## Introduction

Cornelia de Lange Syndrome (CdLS; OMIM# 1227470, 300590, 610759, 614701, and 300882) is a heterogeneous disorder in relation to both patients' phenotype and genotype (1, 2). Alterations in genes encoding subunits or regulators of the cohesin complex, especially those in *NIPBL*, are detected in the majority of CdLS patients. The basic function of the cohesin complex is to control sister chromatids separation during mitosis and meiosis (3). However, abnormal segregation of chromosomes is not observed in the patient's cell lines (4) and cell cycle disorders are not observed in patients with CdLS. More recent studies indicate the involvement of the cohesin complex in other chromatin-related processes, namely in mediating sister chromatid cohesion, chromatin remodeling, transcriptional regulation, and DNA double-strand break repair (5). Current data suggest that these functions of the complex may be impaired in a group of CdLS patients.

Interestingly, alterations in genes, such as *ATRX, KMT2A*, and *TAF6*, involved in the epigenetic modification, chromatin remodeling, and transcriptional regulation pathways are responsible for CdLS-like phenotypes (6). *ATRX* encodes a helicase which regulates the transcription of downstream genes and modifies chromatin structure. *KMT2A* encodes a histone H3 lysine 4 (H3K4) methyltransferase that regulates chromatin-mediated transcription. TAF6 is a component of transcription factor IID (TFIID), which consists of TATA-binding protein (TBP) and 12–14 TBP-associated factors (TAFs) which promote transcriptional initiation. Pathogenic variants in these genes can alter the expression of different genes and lead to abnormalities in multiple body systems, such as those observed in CdLS patients.

It is accepted that mosaic variants in *NIPBL* are frequent in CdLS patients (7). This type of genetic alterations can lead to the different expression of symptoms among patients. Moreover, only very sensitive techniques can allow detecting this specific type of molecular changes.

In this study, comprehensive mutational analysis was performed to determine the prevalence and spectrum of germline and mosaic alterations in genes encoding subunits and regulators of the cohesin complex and three transcriptional regulators in a group of Polish patients with CdLS. Additionally, the impact of intronic variants on mRNA splicing was studied.

## Materials and Methods

### Human Subjects and Sample Collection

A total of 69 CdLS individuals were enrolled in the study, where 39 were females (56,5%), and 30 were males (43,5%). In all patients, CdLS was diagnosed and divided into two groups based on the phenotype according to accepted criteria (8, 9). The first group composed of 33 (47,8%) individuals defined as presenting a classic CdLS phenotype. The second group consisted of 36 (52,2%) individuals with mild CdLS. Of the 39 females, 18 (46,2%) individuals presented the classic phenotype and 21 (53,8%) the mild phenotype. Of the 30 males, 15 (50%) individuals presented classic phenotype and 15 (50%) mild phenotype. To note, 15 previously *NIPBL*-negative patients were included in the study (10).

DNA was successfully extracted from peripheral blood samples of 68 (98,6%) individuals. In addition, buccal swabs were collected from 21 (30,5%) individuals. To confirm a pathogenic status of detected intronic variants, RNA was extracted from peripheral blood samples of 7 (10,1%) individuals. Finally, familial cosegregation was performed in 31 patients' relatives.

The study was approved by the local ethics committee at the Medical University of Gdansk. Parents gave written informed consent for molecular genetic testing of their children.

### DNA Isolation and Mutational Analysis

Genomic DNA was isolated from peripheral blood lymphocytes and oral mucosa epithelial cells using Genomic DNA from blood and Genomic DNA from tissue kits (Macherey-Nagel) according to the manufacturer's protocols.

Mutational analysis was performed using NimbleGen Seqcap EZ HyperCap (Roche Diagnostics) and MiSeq (Illumina). In targeted gene-panel, all genes involved in the cohesin complex were selected. Targeted gene-panel was designed to cover entire coding sequences and 25 bp of flanking introns of *NIPBL, SMC1A, SMC3, RAD21, HDAC8, STAG1, SGOL1, PDS5A, PTTG1, TAF6, ESCO2, WAPAL, CDCA5, KMT2A, DDX11, ESPL1, PDS5B, PLK1, AURKB, ESCO1, MAU2, ATRX, STAG2*, and *RECQL4* genes.

Bioinformatics analysis was performed using IGV (Broad Institute), SeqNext (JSI Medical Systems), and Alamut (Interactive Biosoftware) software. The nomenclature of the alterations was based on the mRNA sequence (Supplementary Table 1) according to the recommendations of the Human Genome Variety Society (11). Variants were classified based on the American College of Medical Genetics' (ACMG) recommendations (12).

In addition, screening for large rearrangements in *NIPBL* was performed using Multiplex Ligation-dependent Probe Amplification technique (P141 and P142, MRC Holland). PCR fragments were analyzed using the 3100 Series Genetic Analyzer (Thermo Fisher Scientific). Data were analyzed using Coffalyser.Net software (MRC Holland).

Detected pathogenic or potentially pathogenic variants were confirmed by independent PCR reactions followed by bidirectional Sanger sequencing. All primer sequences and annealing temperatures are presented in Supplementary Table 2. Electropherograms were analyzed with Sequencher v.10 DNA Software (Gene Codes).

To analyze the effect of detected variants on mRNA splicing, additional blood samples were collected. RNA was extracted using Tempus Spin RNA Isolation Reagent Kit (Thermo Fisher Scientific), and iScript cDNA Synthesis Kit (Bio-Rad) was applied for cDNA synthesis. PCR products were excised from agarose gel and bidirectionally sequenced. For two individuals (CdLS24 and CdLS62), excised PCR products were cloned into a pGEM-T Easy Vector System (Promega) followed by DNA isolation (Plasmid Mini, A&A Biotechnology) and Sanger sequencing. Electropherograms were analyzed with Sequencher v.10 DNA Software (Gene Codes).

## Results

In total, 69 patients were enrolled to the study. DNA extraction from blood samples was successful for 68 individuals (98,6%) while MLPA and deep (NGS) sequencing analysis was performed in 66 (95,7%) and 67 (97,1%) patients, respectively. Large rearrangements of *NIPBL* were not identified in the studied group. However, pathogenic and potentially pathogenic germline substitutions (*n* = 8; 11,6%), duplications (*n* = 4; 5,8%), and deletions (*n* = 2; 2,9%) of *NIPBL* were detected in 14 (20,3%) individuals. Moreover, four germline variants were found in *SMC1A* (*n* = 2; 2,9%) and in *HDAC8* (*n* = 2; 2,9%). In general, germline variants were detected in 18 (26,1%) patients.

**Figure 1:**
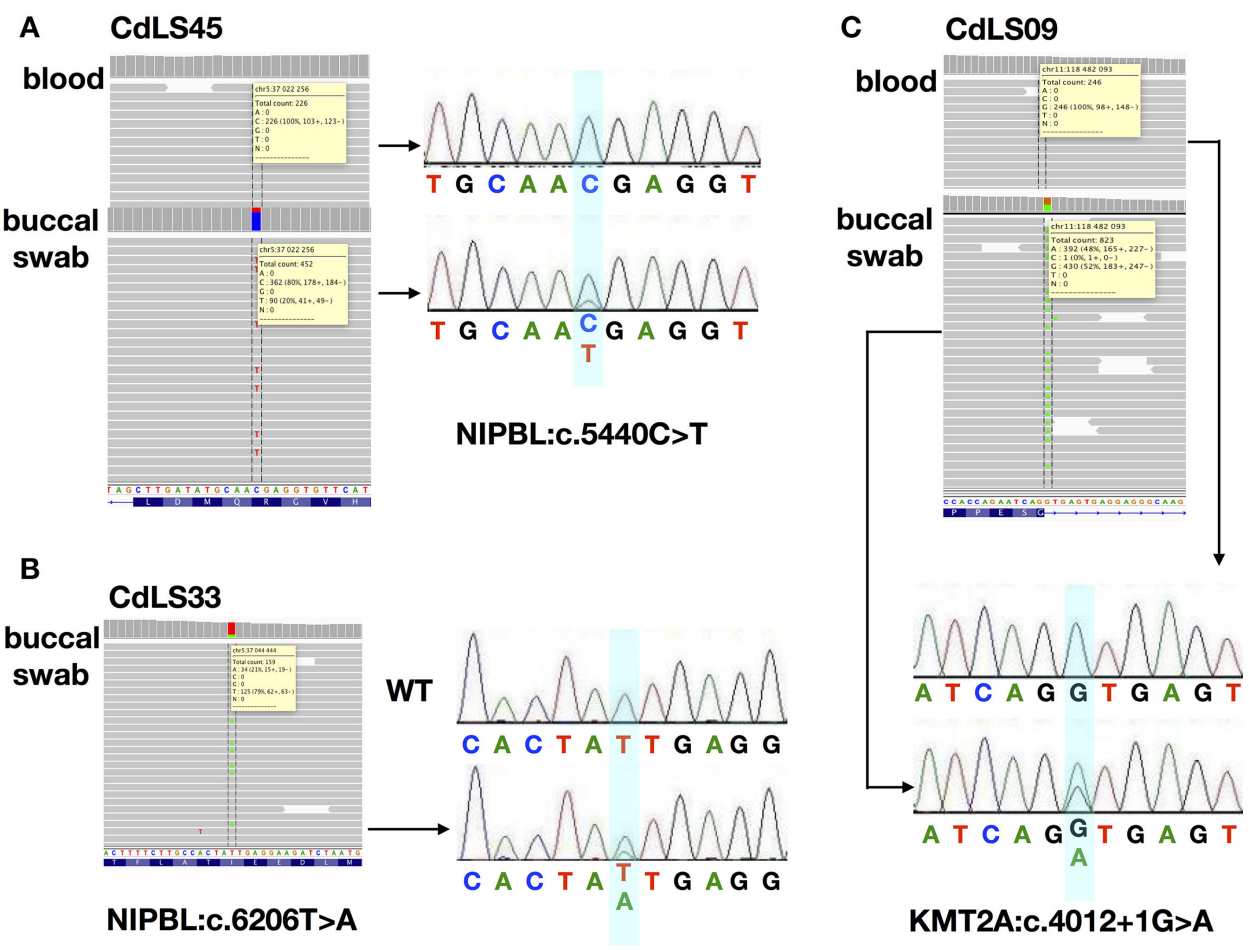
The analysis for the characterization of three mosaic variants. **(A)** NGS analysis and Sanger sequencing chromatograms of gDNA from blood and buccal swab samples of CdLS45 patient (NIPBL:c.5440C>T). **(B)** NGS analysis and Sanger sequencing chromatograms of gDNA from buccal swab samples of CdLS33 patient (NIPBL:c.6206T>A). Sanger sequencing chromatograms of gDNA of WT sample. **(C)** NGS analysis and Sanger sequencing chromatograms of gDNA from blood and buccal swab samples of CdLS09 patient (KMT2A:c.4012+1G>A). Dotted lines in the IGV software and the bluish shading in the chromatograms mark the position if the variants.

**Figure 2:**
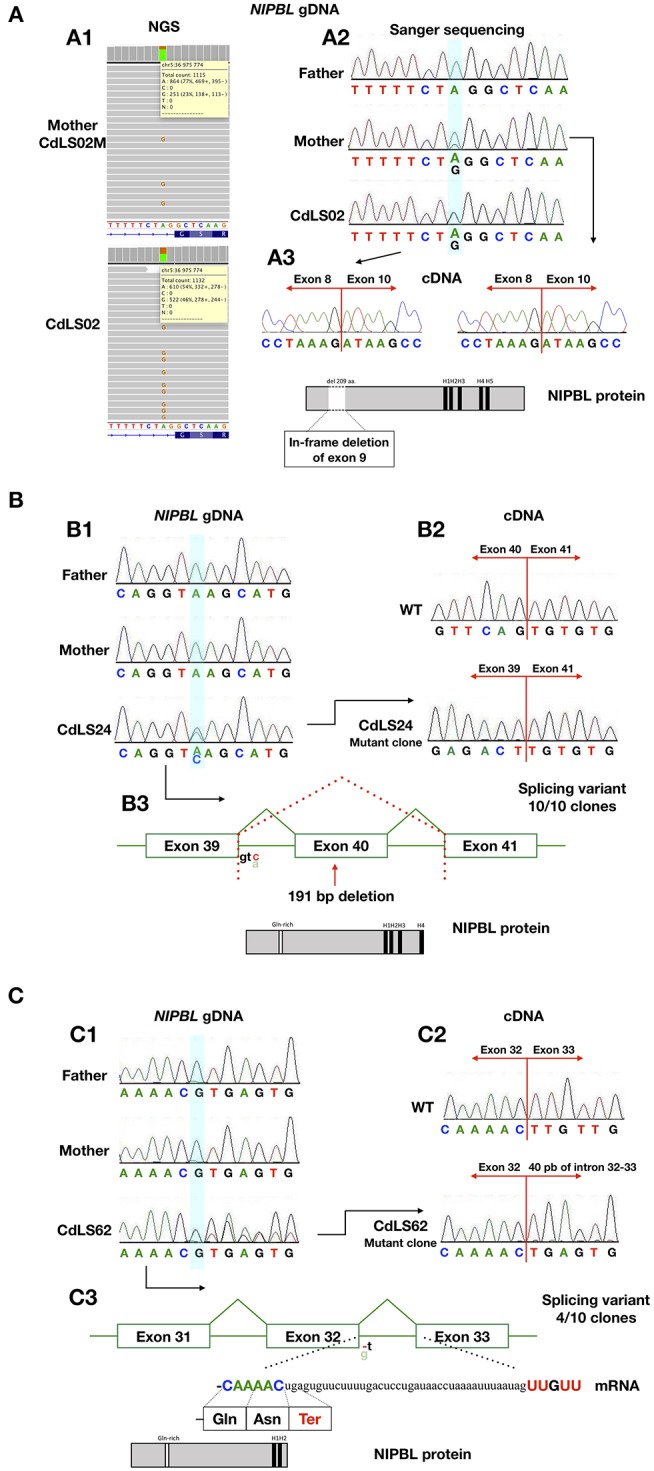
The analysis for the characterization of the splicing variants. **(A)** Analysis of patients CdLS02 and CdLS02M (NIPBL:c.896-2A>G). (A1) NGS analysis of DNA from the mother's blood samples (CdLS02M) and the son (CdLS02), on IGV software. (A2) Chromatograms showing the segregation analyses of gDNA Sanger sequencing. The bluish shading in the chromatograms marks the position of the variant. (A3) Chromatograms showing cDNA Sanger sequencing analysis of the mother (CdLS02M) and the son (CdLS02) showing loss of exon 9 in mRNA sequence. The outcomes of alternations splicing of NIPBL protein alignment of the patients with marked in-frame deletion of exon 9 and loss of Gln-rich domain. **(B)** Analysis of patients CdLS24 (NIPBL:c.6954+3A>C). (B1) Chromatograms showing the segregation analyses of gDNA Sanger sequencing (B2) Chromatograms showing cDNA Sanger sequencing analysis of wild-type and patient (after cloning in the pGEM –T Easy Vector system) showing loss of exon 40 in mRNA sequence. (B3) The outcomes of alternations splicing with loss of exon 40. NIPBL protein alignment exhibits protein truncation and loss of H5 domain. **(C)** Analysis of the patient CdLS (NIPBL:c.5862+1delC). (C1) Chromatograms showing the segregation analyses of gDNA Sanger sequencing. (C2) Chromatograms showing cDNA Sanger sequencing analysis of wild-type and patient (after cloning in the pGEM –T Easy Vector system) showing in-frame insertion of 40 nucleotides of intron 32-33. (C3) The outcomes of alternations splicing with the insertion of 40 nucleotides of intron 32-33. NIPBL protein alignment exhibits protein truncation and loss of H3, H4, and H5 domains.

Only 13 (18,8%) buccal swabs were suitable for deep (NGS) sequencing. In one individual (CdLS33), DNA isolated from buccal swabs was the only available material for the molecular testing. In our cohort, mosaic variants were identified in four (30,8%; 4/13) patients negative for germline alterations. Three (23%; 3/13) mosaic substitutions were detected in *NIPBL* and one (7%; 1/13) in *KMT2A* (Figures 1, 2A). In one patient (CdLS45), c.5440C>T (p.Arg1814Ter) *NIPBL* variant was detected in 20% (90/452) of reads in DNA extracted from the buccal swab sample. This genetic variant was not detected in the blood sample. In another patient (CdLS33), substitution c.6206T>A (p.Ile2069Asn) in *NIPBL* was found in 21% (34/159) of reads of the buccal swab sample. Unfortunately, the blood sample of this patient was not available for the study. In the patient CdLS02M, substitution (c.869-2A>G, p.Gly290_Lys498del) was detected in 23% (251/1115) and 51% (74/148) of reads in DNA extracted from blood and buccal swab samples, respectively (13). Finally, a novel intronic and mosaic variant of *KMT2A* (c.4012+1G>A) was found in 48% (349/823) of reads of DNA extracted from buccal swab sample of patient CdLS09. This variant was not detected in a blood sample of the patient.

RNA was extracted from blood samples of seven (10,1%) individuals. In four cases, cDNA analysis confirmed that detected variants have an impact on RNA, splicing and alter sequences of encoded proteins (Figure 2). The c.869-2A>G *NIPBL* substitution identified in patients CdLS02 and CdLS02M leads to an in-frame exon 9 skipping (r.869_1495del; p.Gly290_Lys498del). Another *NIPBL* variant (c.6954+3A>C) found in CdLS24 patient, resulted in exon 40 skipping (r.6764_6954del; p.Ser2255Leufs^*^20). In the CdLS62 patient, substitution of *NIPBL* c.5862+1delG caused insertion of 40 nucleotides of intron 32 between sequence of exons 32 and 33 (r.5862_5863ins5862+2_5862_41; p.Leu1955Ter). Patients CdLS24 and CdLS62 were previously reported (14). Pathogenicity, predicted by *in silico* analysis, of the *SMC1A* variant (c.1114-3_1114-2delCA), detected in patient CdLS29 was not confirmed by mRNA analysis. This variant was re-classified as benign.

Finally, familial cosegregation analysis was performed in 14 (20,3%) individuals. Only in one case (CdLS02), a *NIPBL* pathogenic variant was detected in the patient and his mother (13).

**Figure 3:**
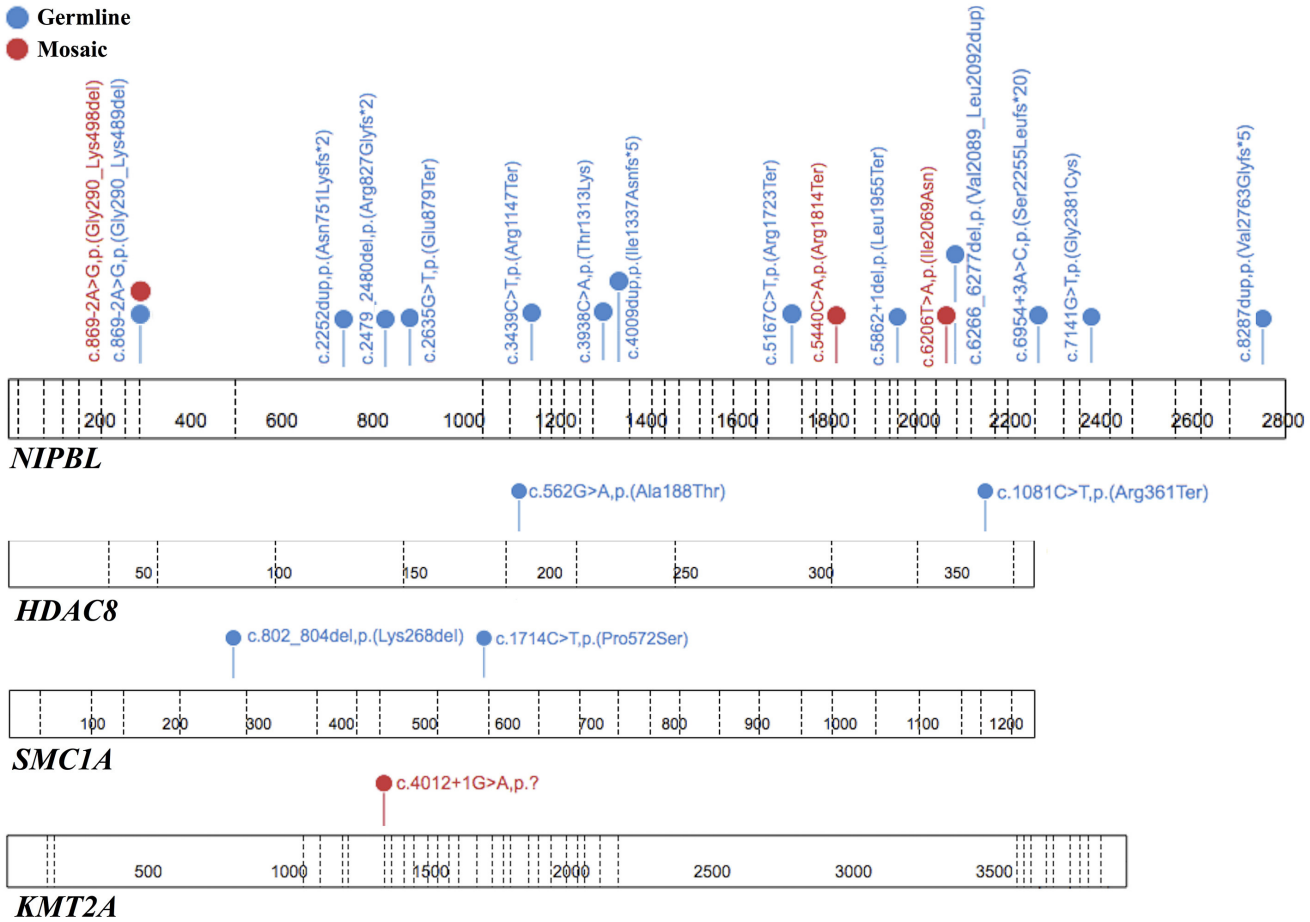
The spectrum of the pathogenic or likely pathogenic variants identified in tested genes. Each variant is unique for one patient. The figure was prepared using the ProteinPaint application (15).

To summarize, both germline and mosaic variants were found in 22/69 (31,9%) patients. All identified variants are presented in Table 1 and Figure 3.

**Table 1:** Pathogenic and likely pathogenic variants detected in the studied group of 69 patients with Cornelia de Lange Syndrome.

**No**.	**Case no**.	**Gender (M/F)**	**Phenotype**	**Exon/intron**	**Variant in corresponding cDNA**	**Predicted effect**	**Classification**	**Coverage [blood sample (%)]/[buccal swab sample (%)]**	**Sanger sequencing**	
									**Mother**	**Father**
***NIPBL***										
1	CdLS02	M	Classic	8	c.869-2A>G	r.[869_1495del];[869_1495 = ] p.(Gly290_Lys498del)	Pathogenic	[522/1132(46%)]/[ND]	c.[869-2A>G];[869-2 = ]	WT
2	CdLS02M	F	Mild	8	c.869-2A>G	r.[869_1495del];[869_1495 = ] p.(Gly290_Lys498del)	Pathogenic	[251/1115(23%)]/[76/148(51%)]	ND	ND
3	CdLS03	F	Mild	29	c.5507G>A	r.[5507g>a];[5507 = ] p.(Gly1836Asp)	Likely pathogenic	[105/221(48%)]/[ND]	WT	WT
4	CdLS04	M	Classic	26	c.5167C>T	p.(Arg1723Ter)	Pathogenic	[153/281(54%)]/[ND]	ND	ND
5	CdLS11	M	Classic	17	c.3938C>A	r.[3938c>a];[3938 = ] p.(Thr1313Lys)	Likely pathogenic	[133/286(47%)]/[ND]	WT	WT
6	CdLS22	F	Classic	12	c.3439C>T	p.(Arg1147Ter)	Pathogenic	[145/314(46%)]/[ND]	WT	WT
7	CdLS24	M	Classic	40	c.6954+3A>C	r.[6764_6954del];[6764_6954 = ] p.(Ser2255Leufs^*^20)	Pathogenic	[98/209(47%)]/[ND]	WT	WT
8	CdLS33	F	Classic	35	c.6206T>A	p.(Ile2069Asn)	Likely pathogenic	[ND]/[34/159(21%)]	WT	WT
9	CdLS42	F	Classic	17	c.4009dup	p.(Ile1337Asnfs^*^5)	Pathogenic	[122/257(47%)]/[ND]	WT	WT
10	CdLS45	F	Classic	29	c.5440C>T	p.(Arg1814Ter)	Pathogenic	[WT]/[90/452(20%)]	ND	ND
11	CdLS53	F	Classic	10	c.2252dup	p.(Asn751Lysfs^*^2)	Pathogenic	[132/324(41%)]/[ND]	WT	WT
12	CdLS54	M	Classic	10	c.2635G>T	p.(Glu879Ter)	Pathogenic	[160/320(50%)]/[ND]	ND	ND
13	CdLS55^*^	F	Classic	36	c.6266_6277dup	p.(Val2089_Leu2092dup)	Likely pathogenic	[7/16(44%)]/[ND]	WT	WT
14	CdLS58	F	Classic	42	c.7141G>T	p.(Gly2381Cys)	Likely pathogenic	[107/258(41%)]/[ND]	ND	ND
15	CdLS62	F	Classic	32	c.5862+1delG	r.[5862_5863ins5862+2_5862+41];[5862_5863 = ] p.(Leu1955Ter)	Pathogenic	[78/165(47%)]/[ND]	WT	WT
16	CdLS63	M	Mild	10	c.2479_2480delAG	p.(Arg827Glyfs^*^2)	Pathogenic	[113/235(48%)]/[ND]	WT	WT
17	CdLS68	F	Mild	47	c.8287dup	p.(Val2763Glyfs^*^5)	Pathogenic	[509/1085(47%)]/[ND]	WT	WT
***HDAC8***										
17	CdLS21	F	Classic	10	c.1081C>T	p.(Arg361Ter)	Pathogenic	[135/271(50%)]/[ND]	WT	WT
19	CdLS65	F	Mild	6	c.562G>A	p.(Ala188Thr)	Pathogenic	[463/966(48%)]/[ND]	WT	WT
	SMC1A	
20	CdLS05	F	Mild	10	c.1714C>T	p.(Pro572Ser)	Likely pathogenic	[211/427(49%)]/[ND]	ND	ND
21	CdLS59	F	Classic	5	c.802_804delAAG	p.(Lys268del)	Likely pathogenic	[153/318(48%)]/[ND]	ND	ND
***KMT2A***										
22	CdLS09	M	Mild	7	c.4012+1G>A	p.?	Pathogenic	[WT]/[392/823(48%)]	ND	ND

Gender: M, male; F, female. ND, no data; WT, wild-type in tested fragments;

* *, TruSightOne*.

## Discussion

Overall, pathogenic and potentially pathogenic variants were found in 22 (31,9%) individuals. Of those, 18 (26,1%) alterations were germline while four (5,8%) were mosaic. No large rearrangements of the *NIPBL* were identified in the patients analyzed. The frequency of alterations detected in this study was lower compared to the previous reports (31,9% vs. 60–80%) (2, 16–18). This low rate of detected alterations can be explained by selection bias since 15 previously *NIPBL*-negative patients were included in the study (10).

It is accepted that genetic variants are more frequently detected in CdLS patients with classic phenotype than in those with a mild phenotype. Indeed, in our studied group of patients, pathogenic and likely pathogenic variants were found in 45,5% (15/33) individuals with classic phenotype and only in 19,4% (7/36) patients with mild CdLS phenotype. The mutation detection rate among patients with classic phenotype is closer to the data reported in the literature (48,5% vs. 60–80%). Therefore, we can speculate that some patients with a mild phenotype may be diagnosed with a genetic syndrome phenotypically similar to CdLS but with a distinct molecular basis.

In CdLS patients negative for germline mutations, mosaic alterations can be detected. Due to the high sensitivity of the method, NGS seems to be the most appropriate technique to identify such variants (17, 19). To date, several patients with mosaic variants in CdLS-related genes have been reported (17, 20, 21). In a group of 44 patients, Huisman et al. detected mosaic variants in *NIPBL* in 10 individuals which accounts for 23% of all identified alterations (7). In the current study, mosaic variants in *NIPBL* have been identified in three out of 17 *NIPBL*-positive patients (17,6%). Two of them had classic and one mild phenotype of CdLS.

Among other tested genes, a mosaic variant was detected only in one patient with mild CdLS phenotype. It was a novel, intronic c.4012+1G>A variant in *KMT2A*. This patient demonstrated CdLS–like phenotype with the pre and postnatal microsomy, some dysmorphic features: long eyelashes, low set ears, thin lips, high arched palate, small hands, and feet with V finger clinodactyly, hirsutism with the excessive hair growth near sacrum, mild developmental delay with autistic-like behavior and speech impairment and feeding difficulties. Parents did not agree for the publication of the photography. *In silico* analysis confirmed a pathogenic character of the identified variant since a change of splice-donor site results in an aberrant transcript. Unfortunately, no further sample of this patient was available for RNA studies. Nevertheless, germline *KMT2A* variant (c.2233C>T, p.Arg745Ter) has been previously described in a patient with an overlapping phenotype of CdLS and Wiedemann-Steiner syndrome (6). *KMT2A* pathogenic variants have been suggested to contribute to the CdLS-like phenotypic spectrum (6). To our knowledge, our patient is the first case of mild CdLS phenotype, and an intronic mosaic variant in the *KMT2A* gene.

Based on the previous study, approximately 20% of *NIPBL* variants are located within intron sequences (19). In this study, three splice site variants have been identified in the *NIPBL* gene in four patients, three with classic CdLS and one with a mild phenotype (mosaic splice site variant). RNA analysis confirmed the pathogenic impact of all variants on the splicing process. In contrast, a splicing variant c.1114-3_1114-2delCA detected in a conserved region of the *SMC1A* gene did not result in an aberrant transcript (patient CdLS29). The actual sequence c.1114-5_1114-4 is identical to the deleted one; therefore splice-acceptor site was not altered nor changed its position. Based on these data, the detected c.1114-3_1114-2delCA variant was re-classified as benign. Splice variants appear to occur more often in patients with a more severe phenotype (22). Nonetheless, few family cases with splice variants and mild phenotype have been reported (23). However, based on our data, such variants in a mosaic stage can also be detected in CdLS patients with a mild phenotype. In our cohort, we were able to identify germline splice variant in the son (CdLS02) of an affected patient (CdLS02M) diagnosed with a mosaic alteration and mild phenotype (13).

In summary, pathogenic variants in selected genes were identified in 22 (31,9%) of 69 CdLS patients. These results indicate that in patients with CdLS, deep (NGS) sequencing techniques should be applied in order to detect a molecular background of the disease. Molecular testing of CdLS patients should allow detecting the presence of mosaic variants, which can be identified in a significant number of individuals. Furthermore, this study showed that simple transcript analysis could be useful to classify pathogenicity of detected intronic variants.

## Conclusion

Next Generation Sequencing methods should be applied to perform molecular testing of patients with CdLS/CdLS-like phenotypes in order to increase the detection rate of all possible genetic variants, including mosaic and splice site variants, which can be detected in this group of individuals.

## Ethics Statement

The study was approved by the Ethical Committee of the Medical University of Gdansk, Poland (NKEBN/395/2014; NKEBN/395-504/2014; NKEBN/395-288/2014). Written informed consent for publication was obtained from patients' parents.

## Author Contributions

JW: classification of the patients in the study. NK and BW: conceived and designed the experiments and analyzed the data. NK: performed the experiments and prepared the manuscript. NK, JW, and BW: manuscript editing. All authors read and approved the final manuscript.

### Conflict of Interest Statement

The authors declare that the research was conducted in the absence of any commercial or financial relationships that could be construed as a potential conflict of interest.

## References

[1] Moss J Penhallow J Ansari M Barton S Bourn D FitzPatrick DR. Genotype-phenotype correlations in Cornelia de Lange syndrome: behavioral characteristics and changes with age. Am J Med Genet A. (2017) 173:1566–74. 10.1002/ajmg.a.38228 28425213

[2] Mannini L Cucco F Quarantotti V Krantz ID Musio A. Mutation spectrum and genotype-phenotype correlation in cornelia de lange syndrome. Hum Mutat. (2013) 34:589–96. 10.1002/humu.22430 24038889 PMC3880228

[3] Drosett D Krantz ID On the molecular etiology of Cornelia de Lange Syndrome. Ann N Y Acad Sci. (2009) 1151:22–37. 10.1111/j.1749-6632.2008.03450.x 19154515 PMC2733214

[4] Revenkova E Focarelli ML Susani L Paulis M Bassi MT Mannini L. Cornelia de Lange syndrome mutations in SMC1A or SMC3 affect binding to DNA. Hum Mol Genet. (2009) 18:418–27. 10.1093/hmg/ddn369 18996922 PMC2722190

[5] Metha GD Kumar S Srivastava S Ghosh SK Cohesin: functions beyond sister chromatid cohesion. FEBS Lett. (2013) 587:2299–312. 10.1016/j.febslet.2013.06.035 23831059

[6] Yuan B Pehlivan D Karaca E Patel N Charng WL Gambin T. Global transcriptional disturbances underlie Cornelia de Lange syndrome and related phenotypes. J Clin Invest. (2015) 125:636–51. 10.1172/JCI77435 25574841 PMC4319410

[7] Huisman S Redeker E Maas S Mannens M Hennekam R. High rate of mosaicism in individuals with Cornelia de Lange syndrome. J Med Genet. (2013) 50:339–44. 10.1136/jmedgenet-2012-101477 23505322

[8] Kline AD Krantz ID Sommer A Kliewer M Jackson LG FitzPatrick DR. Cornelia de Lange syndrome: clinical review, diagnostic and scoring systems, and anticipatory guidance. Am J Med Genet A. (2007) 143A:1287–96. 10.1002/ajmg.a.31757 17508425

[9] Kline AD Moss JF Selicorni A Bisgaard AM Deardorff MA Gillett PM. Diagnosis and management of Cornelia de Lange syndrome: first international consensus statement. Nat Rev Genet. (2018) 19:649–66. 10.1038/s41576-018-0031-0 29995837 PMC7136165

[10] Kuzniacka A Wierzba J Ratajska M Lipska BS Koczkowska M Malinowska M. Spectrum of *NIPBL* gene mutations in Polish patients with Cornelia de Lange syndrome. J Appl Genet. (2013) 54:27–33. 10.1007/s13353-012-0126-9 23254390 PMC3548104

[11] den Dunnen JT Dalgleish R Maglott DR Hart RK Greenblatt MS McGowan-Jordan J. HGVS recommendations for the description of sequence variants: 2016 update. Hum Mutat. (2016) 37:564–9. 10.1002/humu.22981 26931183

[12] Richards S Aziz N Bale S Bick D Das S Gastier-Fister J. Standards and guidelines for the interpretation of sequence variants: a joint consensus recommendation of the American College of Medical Genetics and Genomics and the Association for Molecular Pathology. Genet Med. (2015) 17:405–24. 10.1038/gim.2015.30 25741868 PMC4544753

[13] Krawczynska N Kuzniacka A Wierzba J Parenti I Kaiser FJ Wasag B. Mosaic intronic NIPBL variant in a family with Cornelia de Lange syndrome. Front Genet. (2018) 9:255. 10.3389/fgene.2018.00255 30057591 PMC6053508

[14] Krawczynska N Wierzba J Jasiecki J Wasag B. Molecular characterization of two novel intronic variants of NIPBL gene detected in unrelated Cornelia de Lange syndrome patients. BMC Med Genet. (2019) 20:1. 10.1186/s12881-018-0738-y 30606125 PMC6318863

[15] Zhou X Edmonson MN Wilkinson MR Patel A Wu G Liu Y. Exploring genomic alteration in pediatric cancer using ProteinPaint. Nat Genet. (2016) 48:4–6. 10.1038/ng.3466 26711108 PMC4892362

[16] Huisman S Mulder PA Redeker E Bader I Bisqaard AM Brooks A. Phenotypes and genotypes in individuals with SMC1A variants. Am J Med Genet A. (2017) 173:2108–25. 10.1002/ajmg.a.38279 28548707

[17] Pozojevic J Parenti I Graul-Neumann L Ruiz Gil S Watrin E Wendt KS. Novel mosaic variants in two patients with Cornelia de Lange syndrome. Eur J Med Genet. (2017) 61:680–4. 10.1016/j.ejmg.2017.11.004 29155047

[18] Nizon M Henry M Michot C Baumann C Bazin A Bessieres B. A series of 38 novel germline and somatic mutations of NIPBL in Cornelia de Lange syndrome. Clin Genet. (2016) 89:584–9. 10.1111/cge.12720 26701315

[19] Pie J Puisac B Henandez-Marcos M Teresa-Rodrigo ME Gil-Rodriguez M Baquero-Montoya C. Special cases in Cornelia de Lange syndrome: the Spanish experience. Am J Med Genet C Semin Med Genet. (2016) 172:198–205. 10.1002/ajmg.c.31501 27164022

[20] Ansari M Poke G Ferry Q Williamson K Aldridqe R Meynert AM. Genetic heterogeneity in Cornelia de Lange syndrome (CdLS) and CdLS- like phenotypes with observed and predicted levels of mosaicism. J Med Genet. (2014) 51:659–68. 10.1136/jmedgenet-2014-102573 25125236 PMC4173748

[21] Parenti I Gervasini C Pozojevic J Wendt KS Watrin E Azzollini J. Expanding the clinical spectrum of the ‘*HDAC8*-phenotype' – implications for molecular diagnostics, counseling and risk prediction. Clin Genet. (2016) 89:564–73. 10.1111/cge.12717 26671848

[22] Teresa-Rodrigo ME Eckhold J Puisac B Pozojevic J Dalski A Gil-Rodriquez MC. Functional characterization of NIPBL physiological splice variants and eight splicing mutations in patients with Cornelia de Lange syndrome. Int J Mol Sci. (2014) 15:10350–64. 10.3390/ijms150610350 24918291 PMC4100155

[23] Masciadri M Ficcadenti A Milani D Cogliati F Divizia MT Larizza L. Recurrence and familial inheritance of intronic *NIPBL* pathogenic variant associated with mild CdLS. Front Neurol. (2018) 9:967. 10.3389/fneur.2018.00967 30538663 PMC6277459

