# Supplementary material for: Genetic Mosaicism in a Group of Patients With Cornelia de Lange Syndrome
Source: Front Pediatr. 2019 May 15;7:203. doi: 10.3389/fped.2019.00203 (PMC6530423; doi:10.3389/fped.2019.00203)
Supplement: Supplementary file 1 [file Data_Sheet_1.docx]

Supplementary Material

Genetic mosaicism in a group of patients with Cornelia de Lange Syndrome.

Natalia Krawczynska^1,2^, Jolanta Wierzba^3^, Bartosz Wasag^1,2^*

^1^ Department of Biology and Medical Genetics, Medical University of Gdansk, Gdansk, Poland

^2^ Laboratory of Clinical Genetics, University Clinical Centre, Gdansk, Poland

^3^Department of General Nursery, Medical University of Gdansk, Gdansk, Poland

***Corresponding author:**

Bartosz Wasag, Ph.D.

[bwasag@gumed.edu.pl](mailto:bwasag@gumed.edu.pl)

**Table 1.** List of all tested genes in NGS analysis and numbers of transcripts.

| Gene | Transcript |
| --- | --- |
| NIPBL | NM_113433.3 |
| SMC1A | NM_006306.2 |
| SMC3 | NM_005445.3 |
| RAD21 | NM_006265.2 |
| HDAC8 | NM_018486.2 |
| STAG1 | NM_005862.2 |
| SGOL1 | NM_001012410.4 |
| PDS5A | NM_001100399.1 |
| PTTG1 | NM_001282382.1 |
| TAF6 | NM_005641.3 |
| ESCO2 | NM_001017420.2 |
| RECQL4 | NM_004260.3 |
| WAPAL | NM_015045.3 |
| CDCA5 | NM_080668.3 |
| KMT2A | NM_012308.2 |
| DDX11 | NM_152438.1 |
| ESPL1 | NM_012291.4 |
| PDS5B | NM_015032.3 |
| PLK1 | NM_005030.5 |
| AURKB | NM_001284526.1 |
| ESCO1 | NM_052911.2 |
| MAU2 | NM_015329.3 |
| ATRX | NM_000489.3 |
| STAG2 | NM_001042749.2 |

**Table 2. List of primers and annealing temperatures.**

| Case no | Exon/ intron | Forward (5’-3’) | Revers (5’-3’) | Annealing (C) |
| --- | --- | --- | --- | --- |
| NIPBL - DNA | | | | |
| CdLS02/ CdLS02M | 8-9A | CACAACTGTTACTTCTATCGA | TGTCCTGGGTACTGCACA | 63-56 |
| CdLS03/ CdLS45 | 28-29 | ACAAAAATGTCAATGTT | TTTTTCTAACTTAACACTTT | 51 |
| CdLS04 | 26 | CATCAAGCTCAAGTCTGTCTAA | ATAAATGTAAACTGTTTTCTCCTT | 63-56 |
| CdLS11/ CdLS42 | 17 | TCCACCAGTGAAAATCAAATC | TTTTGGTGCCATTTTAAGTCC | 68-61 |
| CdLS20 | 9B | AACTTACATTAAGACTTTCTC | ATGATGAAGTGGTAATAATGAG | 63-56 |
| CdLS22 | 12 | TTGTCATGGGGATTTGCTTC | CTTAAACGATAATCACTGCAC | 63-56 |
| CdLS24 | 40 | GTAAAGTTAGTATAGGTGCTC | AGTGCTGCTGCCATAAAACA | 63-56 |
| CdLS33/ CdLS55 | 35-36 | ATATAGTTTCTTTTCAGGTTTTGGAT | GCAGAATAATTTTAAAACCCTGGTA | 68-61 |
| CdLS54 | 10E | TGAATCAGAGCGACATCGAG | CCCCCAACAAATAACTTGGAA | 63-56 |
| CdLS58 | 42 | TGAAGCTAGCCTCAGAATGTAA | ACTTAATGAAGTTACTATCATATAC | 63-56 |
| CdLS62 | 32 | GGCTAAAGCATAACAAAAGTATATT | AATAAATTTTCCCTACCAAAAGAA | 63-56 |
| CdLS63/ CdLS53 | 10D | AGTGATGGGCATCCTGAAAC | CTTCGAACCCTAGACTGATC | 63-56 |
| CdLS68 | 47 | GCGTCAAGGGATTAAAAGCA | ATGTTTGCCCAACATTTCCTTT | 63-56 |
| HDAC8 - DNA | | | | |
| CdLS21 | 10 | TGTGGTTGGTTTGGAGGTTT | ATTTAAGGAAGCCCCCACTG | 60 |
| CdLS65 | 6 | CCTCCTTCTCACAGCTGCTT | TTGCATAGGATTGTGGCAGT | 59 |
| SMC1A | | | | |
| CdLS05 | 10 | CTGTCCCCCTTTCCATACTG | TCCAGTACTGAGCCTGTCCA | 62 |
| CdLS29 | 6 | CTGGGAACCCTCCCTTCTT | TCTGTGGACCATCATCCCTA | 58 |
| CdLS59 | 5 | TTTTCTGGGTGAAGGAGTGG | ATAAACAGCACGGCCTCTTG | 58 |
| KMT2A - DNA | | | | |
| CdLS09 | 7 | CCGTCGAGGAAAAGAGTGAA | GAGAGCTGATTTTCTTGAACTGAA | 58 |
| NIPBL - cDNA | | | | |
| CdLS03 | 27-31 | TTGATATTTATTTGACACAGATCCT | GAATGTTTCATTTACTAATTTCTTAA | 60 |
| CdLS11 | 17 | GGTATAATGGATAAGCTTTC | CCCATAGATGAAACCTGAA |  |
| CdLS20 | 9B | GACTTTCTCGTGTAAGGTCTTCAG | TCTGTTGTTGATTCTGTACCACA |  |
| CdLS02/ CdLS02M | 7-10 | TGAGAGTCCACTCTTCCTGC | TGAGAGTCCACTCTTCCTGC | 60 |
| CdLS24 | 38-42 | AAGCTATCATTGGTCTAGGATTT | AGTCACGTCTGTTTTTGCTG | 55 |
| CdLS62 | 30-35 | ATGAAGAGGGCATTAAGAAATT | GAAATCATTTTGCGTACTAC | 54 |
| SMC1A - cDNA | | | | |
| CdLS29 | 4-09 | CAGGAGAAAGAAGAGGCTGA | TCTTCTAGGGACTGCTTGCT | 58 |
|  |  |  |  |  |
